# Depression Self-Care Apps’ Characteristics and Applicability to Older Adults: Systematic Assessment

**DOI:** 10.2196/56418

**Published:** 2025-02-21

**Authors:** Ruoyu Yin, Dakshayani Rajappan, Laura Martinengo, Frederick H F Chan, Helen Smith, Konstadina Griva, Mythily Subramaniam, Lorainne Tudor Car

**Affiliations:** 1 Lee Kong Chian School of Medicine Nanyang Technological University Singapore Singapore Singapore; 2 School of Social Sciences Nanyang Technological University Singapore Singapore Singapore; 3 Centre for Behavioural and Implementation Sciences Interventions Yong Loo Lin School of Medicine National University of Singapore Singapore Singapore; 4 School of Medicine Keele University Staffordshire United Kingdom; 5 Research Division Institute of Mental Health Singapore Singapore; 6 Department of Primary Care and Public Health School of Public Health, Faculty of Medicine Imperial College London London United Kingdom

**Keywords:** older adults, elder, elderly, mental health, mental illness, mental disorders, mHealth, mobile health, mobile application, app, application, smartphone, depression, self-care, mobile apps, systematic assessment, assessment, effectiveness, self-care, Android app, mental health apps, mobile interventions, behaviour, therapy, mood monitoring, adaptations, online communities, impairments

## Abstract

**Background:**

Depression affects 32% of older adults. Loneliness and social isolation are common risk factors for depression in older adults. Mobile apps can connect users and are also effective in depression management in the general population. However, older adults have specific needs in terms of the content of depression self-care interventions and their accessibility. It remains unknown whether existing apps for depression self-care are applicable to older adults.

**Objective:**

The initial aim of this assessment was to systematically identify interactive depression self-care apps specifically designed for older adults. As we did not find any, we assessed the applicability of existing depression self-care apps to the needs of older adult users.

**Methods:**

Using an established app assessment methodology, we searched for Android and iOS interactive mental health apps providing self-care for depression in English and Chinese in the 42Matters database, Chinese Android app stores, and the first 10 pages of Google and Baidu. We developed an assessment rubric based on extensive revision of the literature. The rubric consisted of the following sections: general characteristics of the apps (eg, developer, platform, and category), app content (eg, epidemiology and risk factors of depression in older adults, techniques to improve mood and well-being), and technical aspects (eg, accessibility, privacy and confidentiality, and engagement).

**Results:**

We identified 23 apps (n=19, 82.6%, English and n=4, 17.4%, Chinese apps), with 5 (21.7%) iOS-only apps, 3 (13%) Android-only apps, and 15 (65.2%) apps on both platforms. None specifically targeted older adults with depression. All apps were designed by commercial companies and were free to download. Most of the apps incorporated cognitive behavior therapy, mood monitoring, or journaling. All but 3 (13%) apps had a privacy and confidentiality policy. In addition, 14 (60.9%) apps covered depression risk factors in older adults, and 3 (13%) apps delivered information about depression epidemiology in older adults via a chatbot. Furthermore, 17 (73.9%) apps mentioned other topics relevant to older adults, such as pain management, grief, loneliness, and social isolation. Around 30% (n=7) of the apps were supported by an online forum. Common accessibility issues included a lack of adaptations for users with visual or hearing impairments and incompatibility with larger font sizes in the phone settings.

**Conclusions:**

There are no depression apps developed specifically for older adults. Available mobile apps have limited applicability to older adults in terms of their clinical and technical features. Depression self-care apps should aim to incorporate content relevant to older adults, such as grief and loss; include online communities; and improve accessibility to adapt to potential health impairments in older adults.

## Introduction

The population is aging worldwide, and this demographic shift is coupled with higher depression incidence rates in older adults compared to younger populations [1]. China, for example, had 254 million adults over 60 years old in 2019 [2], with a growing incidence of mental disorders and a high risk of suicide [3-5]. In 2020, the number of older adults in the United States was 55.8 million, representing 16.8% of the total population [6]. It was estimated that 8 million older adults in the United States aged 65 years and above had depression, and depression affects 32% of older adults worldwide [7,8]. Older adults with depression are more likely to have comorbidities, worse sleep, decreased quality of life, and poorer self-perceived health compared to those without depression [9,10]. There are many barriers to older adults seeking mental health treatment, including low awareness, which may lead to a poor prognosis [11]. There is a need for effective, scalable, accessible, and affordable mental health interventions that are tailored to the needs of older adults. Digital health interventions, particularly those delivered via mobile phones, have the potential to address this burden of mental illness in older adults.

Older adults are increasingly using mobile phones, which facilitates using mobile apps to improve their mental health. In the United States, the ownership of smartphones between 2017 and 2022 in adults aged 65 years and above increased from 40% to 65% [12]. In China, over a third of the internet users in 2021 were adults aged 50 years or older [13]. The need to minimize in-person contact during the COVID-19 pandemic also boosted the use of digital technology in older adults. For example, in Singapore, more than half of 55-75-year-olds used social media platforms or the internet to search for news of the pandemic [14]. Mobile apps also provide users with an opportunity to stay connected and may potentially mitigate loneliness and social isolation [15], which are common risk factors for depression in older adults [16].

Contrary to some common beliefs, older adults are becoming increasingly tech-savvy, with some preferring to use digital health tools for mental health treatment over face-to-face treatment [17-21]. Furthermore, digital mental health interventions can help reduce the long waiting times for counseling and avoid the time and financial costs of travel from rural areas [22]. In addition, the privacy provided by digital mental health tools may reduce the potential stigma and shame, which is common among older adults [22-26]. Mental health mobile apps have been shown to be effective in the treatment of depression in the general population [27,28]. Although there were mental health apps specifically targeting the youth [29], it remains unknown whether there are apps that cater for older adults’ specific needs in terms of mental health content and digital accessibility [30,31]. For example, worldwide, around 51% of older adults aged 60 years and above have multimorbidity [32] and would benefit from content on physical health, cognitive decline, stress, aging, and isolation [33,34]. They may also need bigger fonts, and louder and clearer audio to address potential visual or hearing impairments [35].

Therefore, the initial aim of this assessment was to systematically identify interactive depression self-care apps specifically designed for older adults. As we did not find any, we assessed the applicability of existing depression self-care apps to the needs of older adult users. We focused on English and Chinese app markets as they represent 31% of the world population [36].

## Methods

### Study Design

We used an app assessment methodology that has been extensively used in previous papers aiming to systematically assess other types of apps [37-40]. The assessment methodology derives from systematic review methodology as it includes a systematic search for apps with a clear mention of the search strategy and databases searched, clear inclusion/exclusion criteria, 2-step screening for eligible apps, and systematic data extraction using a predefined data extraction form. Our method was mostly aligned with a guide on the systematic review and evaluation of mobile health apps [41].

### Search Strategy

We searched for apps in English and Chinese in the 2 leading app markets, Android and iOS, in November-December 2022. We searched Apple’s App Store for English and Chinese apps and Android apps in English using 42Matters, a comprehensive proprietary app database. As Google Play is unavailable in mainland China, we searched the most popular Android Chinese app stores, used by more than 81% of mobile phone users, including Tencent App Store, Huawei App Market, MIUI App Store, Oppo Software Store, VIVO App Store, 360 Mobile Assistant, Baidu Mobile Assistant, MM App Store, PP Assistant, and the Wandoujia platform [42]. In addition, we screened the first 10 pages on popular English and Chinese search engines (Google and Baidu) for any other potentially relevant depression self-care–focused mobile apps [43]. Search terms for apps in English included “mental health,” “mindfulness,” “cognitive behavioral therapy,” “CBT,” “psychotherapy,” “online therapy,” “mood,” “depress,” “anxiety,” “sadness,” “melancholia,” “worry,” and “counseling.” When we combined the aforementioned search terms with “older adults,” “elderly,” “ageing,” or “geriatric” using the AND Boolean operator, no results were returned on 42Matters. Therefore, we decided to broaden the search strategy and manually capture aging-related content in the apps’ descriptions, if any. The search terms for Chinese apps included 抑郁 OR 焦虑 OR 心理健康 OR 心理咨询 OR 行为疗法 OR 精神健康 OR 正念 OR 自杀 OR 悲伤.

### Eligibility Criteria

Textbox 1 shows the inclusion and exclusion criteria of the apps. We included interactive mental health apps that explicitly mentioned depression, were free or required one-time payment to download, and targeted older adults or the general population. Popular apps were included because users are more likely to encounter and use them [44]. As there was no consensus on the criteria related to popularity, previous studies have adopted the strategy of excluding apps with less than 10 [45] or 5 [43,46] reviews. Meanwhile, highly downloaded apps were defined as those with 100,000 or more downloads in Android markets [47]. Therefore, Android apps with ≥100,000 downloads on Google Play or Chinese Android app markets and iOS apps with ≥10 reviews on Apple’s App Store were included. Apps available on both platforms were included if their number of downloads was ≥100,000 in Android markets and they had ≥10 reviews on Apple App Store. Apps that (1) targeted children, adolescents, or young adults; (2) focused exclusively on meditation or mindfulness, positivity, affirmations, or well-being; (3) had technical issues; (4) only allowed consultations with health care providers; or (5) had <100,000 downloads on Google Play or Chinese Android app markets or <10 reviews on Apple’s App Store were excluded.

**Inclusion and exclusion criteria.**

**Inclusion criteria:**

- Targeted older adults or the general population
- Explicitly provided depression self-care
- Included interactive features (ie, “allowing information to be passed continuously and in both directions between a computer or other device and the person who uses it” [47,48])
- Were in English or Chinese
- Were available on Google Play, Chinese Android stores, or Apple’s App Store
- Were found in the following app store categories: health and fitness, lifestyle, and medical
- Were released or updated from May 2021 onwards
- Were free or paid to download
- Had ≥100,000 downloads on Google Play or Chinese Android app markets and ≥10 reviews on Apple’s App Store

**Exclusion criteria:**

- Targeted children, adolescents, or young adults
- Focused exclusively on meditation or mindfulness, positivity, affirmations, or well-being in general
- Had technical issues and could not be used after 2 attempts, required an access code provided by a health care institution or insurance company, or had been removed from the app store at the time of the assessment
- Only allowed online consultations with health care providers
- Had <100,000 downloads on Google Play or Chinese Android app markets or <10 reviews on Apple’s App Store

### App Screening

In the first round of screening, 1 reviewer (author RY) followed the inclusion and exclusion criteria in Textbox 1 to manually screen the apps on ASReview, a screening tool using machine learning algorithms [49] that has been increasingly used in systematic reviews [50-53]. After each decision (include/exclude) by the reviewer, ASReview applied machine learning models (term frequency–inverse document frequency method as the feature extractor and logistic regression as the classifier) and actively sorted the rest of the apps according to their relevance, from the most relevant to the least relevant. As such, ASReview facilitated screening by allowing the reviewer to screen a subset of the total apps. As shown in prior simulation studies, ASReview was able to identify 95% of all relevant records while screening between 8% and 33% of the total records [49]. Since there is no consensus on the stopping rule [54], the reviewer in this study predefined the stopping criteria as screening one-third (33.3%) of the total apps [49]. The apps were then downloaded to an iPhone 13 (iOS 16.1.1), a Samsung A52s (Android 13, One UI 5.1), and 2 of 3 reviewers (authors RY, DR, and FC) independently and in parallel evaluated eligibility. Apps available on both iOS and Android platforms were counted as one app in the assessment.

### Assessment Rubric Development, Data Extraction, and Analysis

To develop an assessment rubric for content relating to older adults, we searched the following keywords on Google Scholar and PubMed: aging, depression clinical practice guidelines, geriatric depression, psychoeducation or psychotherapy for older adults, and accessibility guidelines. We also conducted a qualitative systematic review of older adults’ views and experiences with using digital mental health interventions [55] and a scoping review of experts’ opinions on digital mental health interventions for older adults [56]. Our scoping review confirmed the essential content and features for older adults, including therapeutic approaches, relevant topics, personalization, and accessibility.

Table 1 shows the sources of evidence used in the development of the assessment rubric. The sources included established clinical practice guidelines [57-60], systematic and scoping reviews [61-64], academic books [65-69], a systematic assessment of mental health apps [70], and guidelines and checklists of technical features [71-73], including the Mobile Application Rating Scale (MARS), the mobile Health On the Net Code (mHONcode), and the World Wide Web Consortium (W3C) guidelines for accessibility of content on mobile phones.

Both MARS and mHONcode were designed to evaluate apps’ technical features, while neither of them was specifically designed to assess apps’ applicability to older adults. Furthermore, most of the included apps have been evaluated using these scales elsewhere [45,75-77]. However, existing app assessments have seldom evaluated accessibility in detail. Thus, in this study, we focused on assessing app accessibility using the W3C guidelines, and we used a few items from MARS and mHONcode that do not overlap with the W3C guidelines and are directly relevant to older adults, as indicated in a systematic review on the views and experiences of older adults with digital mental health interventions [55]. According to our review, engagement with the target group (older adults), the quality/resolution of graphics with regard to aesthetics, information credibility, and the evidence base from MARS are relevant and important to older adults [78-80]. In addition, older adults often have concerns about privacy and confidentiality when using digital mental health tools [23,33,78,80-84]. Therefore, we supplemented the W3C guidelines with 3 groups of items from MARS (target group, aesthetics, and information) and 2 items from mHONcode (privacy and confidentiality).

The following information about each app was recorded using a data extraction form (Multimedia Appendix 1) with 3 sections:

- General features of apps, including their description and category in the app store, developers, ratings, target user group, regions, languages, privacy policy, total downloads in Android markets, and cost of download and subscription. Ratings of apps ranged from 1 to 5, with higher scores indicating users’ positive perceptions.
- Content relevant to older adults, which consisted of the following 6 sections: symptoms and natural history of depression in older adults (11 binary questions with yes/no answers and 3 open-ended questions), screening of depression (7 binary questions and 5 open-ended questions), self-care techniques (2 binary questions and 4 open-ended questions), personalization (5 binary questions and 1 open-ended question), human involvement (3 binary questions and 1 open-ended question), and other functions (6 binary questions and 2 open-ended questions).
- Technical features, including the W3C guidelines for accessibility of content on mobile phones [35,73], supplemented by 5 items covering engagement, aesthetics, and information from MARS [71,85] and 2 items on privacy and confidentiality from mHONcode [72]. The W3C guidelines consisted of 4 principles: perceivable (6 binary items), operable (6 items), understandable (7 items), and robust (4 items).

For every item except MARS, a “yes” was assigned 1 point. Each app was independently evaluated by 2 of the 3 reviewers working in parallel (RY, DR, and FC). Assessments were then compared from September to October 2023, and disagreements were discussed until consensus was reached. We used each app for at least 15 minutes to ensure interaction with all app features. Data were recorded in a Microsoft Excel worksheet and summarized. We calculated the percentage of each app’s score over its total score. Narrative data synthesis was used to present our findings.

**Table 1:** Evidence base of the assessment rubric.

Type of evidence	Details/topics
Clinical practice guidelines	The National Institute for Health and Care Excellence in the UK [57] The American Psychiatric Association [74] The Royal Australian and New Zealand College of Psychiatrists [60] The Ministry of Health in Singapore The Japanese Society of Mood Disorders [58] The Indian Psychiatric Society [59]
Systematic reviews or scoping reviews	Mobile health apps for older adults [63] Depression risk factors in older adults [64] Influencing factors of digital technology usage among older adults [61,62] Older adults’ views and experiences of digital mental health interventions [55] Experts’ opinions on digital mental health interventions for older adults [56]
Academic books	Geropsychology [67] Psychotherapy with older adults [69] Aging [68] CBT^a^ [65,66]
Systematic assessment of apps	Self-guided CBT apps for depression [70]
Guidelines and checklists of technical features	MARS^b^ [71] W3C^c^ guidelines for accessibility of content on mobile phones [35,73] mHONcode^d^ [72]

^a^CBT: cognitive behavioral therapy.

^b^MARS: Mobile Application Rating Scale.

^c^W3C: World Wide Web Consortium.

^d^mHONcode: mobile Health On the Net Code.

### Ethical Considerations

Ethical approval was not required for this systematic assessment of mobile apps as no human participants or personal data were involved.

## Results

### Identification of Apps for Depression Self-Care

**Figure 1:**
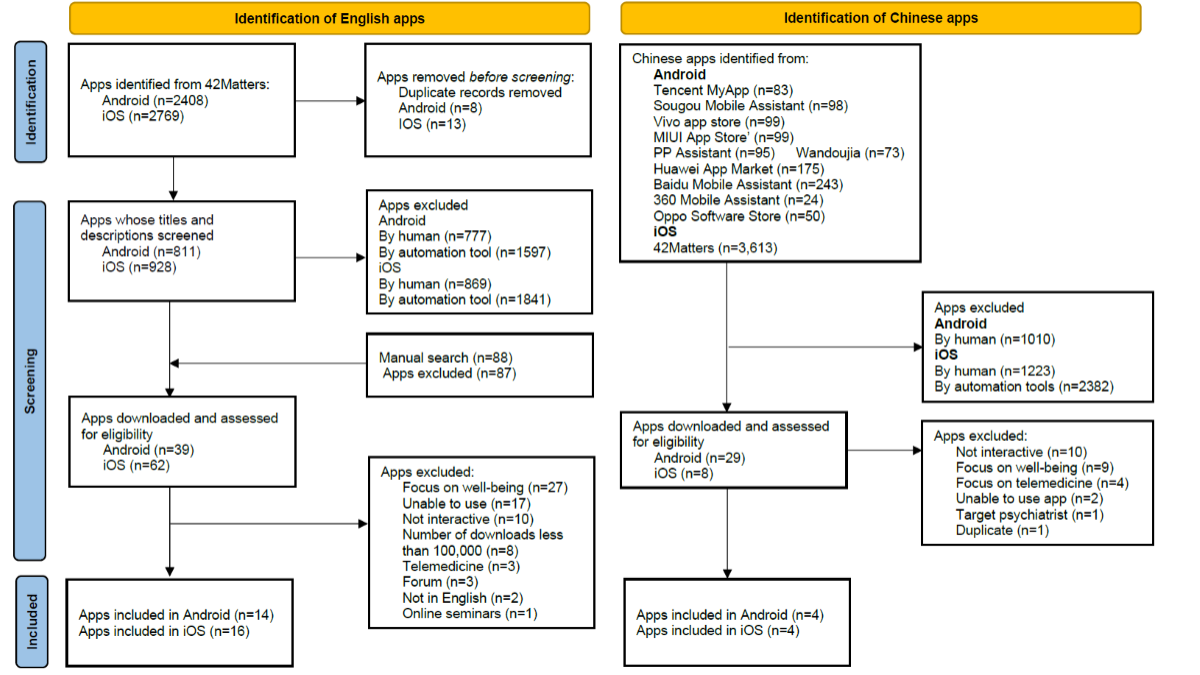
Flowchart of app screening.

We identified 5177 English apps (n=2408, 46.5%, on Android and n=2769, 53.5%, on iOS) in 42Matters and 4652 Chinese apps (n=1039, 22.3%, on Android and n=3613, 77.7%, on iOS) in 42Matters and Chinese app stores. One additional English app, Happify, was identified on Google and none from the Baidu search engine. After title and description screening, 101 (2%) English apps (n=39, 38.6%, on Android and n=62, 61.4%, on iOS) and 37 (0.8%) Chinese apps (n=29, 78.4%, on Android and n=8, 21.6%, on iOS) were downloaded to examine eligibility. We included 23 (16.8%) apps for systematic assessment (n=19, 82.6%, English apps and n=4, 17.4%, Chinese apps. In addition, 15 (65.2%) apps were available on both iOS and Android platforms, 5 (21.7%) on the iOS platform, and 3 (13%) on Android platforms (Figure 1).

### Basic Characteristics of the Included Apps

Table 2 shows the basic characteristics of the included apps. All 23 apps were developed by commercial companies and were free to download. Most of the apps (n=20, 87%) were in the health and fitness category in the app stores, and 3 (13%) were in the medical category. Of the 18 (78.3%) apps available on Android platforms, 8 (44.4%) had been downloaded more than 1 million times; unfortunately, Apple does not provide iOS download data. In addition, 16 (69.6%) apps had in-app purchases for subscriptions or courses or counseling services. Of the 19 (82.6%) English apps, 4 (21.1%) were available in other languages (Chinese, German, French, Italian, Japanese, Portuguese, Spanish, Japanese, and Korean). One of the four Chinese apps supported English and Arabic.

Table 3 shows the ratings of the apps on each platform. Most of the apps available on iOS (n=19, 95%) had a rating of 4-5, indicating good user experience in general, while 1 (5%) app had a rating of 3-4. Similarly, 2 (11.1%) of the 18 apps available on Android had a rating of 3-4, 13 (72.2%) had a rating of 4-5, while ratings were unavailable for 3 (16.7%) apps.

**Table 2:** Basic characteristics of the mental health apps included in this study (N=23).

Basic characteristics		Apps, n (%)
**Affiliation**		
	Commercial	23 (100.0)
	Others (academic institution, government, nongovernmental organization)	0
**Category in the app store**		
	Health and fitness	20 (87.0)
	Medical	3 (13.0)
**Number of downloads on Android markets**		
	100,000-1 million	10 (55.6)
	>1 million	8 (44.4)
**Cost**		
	Free	7 (30.4)
	Free with in-app purchase	16 (69.6)
	Paid	0
**Primary language**		
	English	19 (82.6)
	Chinese	4 (17.4)

**Table 3:** Ratings and number of ratings for the apps included in this study.

Rating details			iOS apps (n=20)		Android apps (n=18)
**Ratings**					
	1-3	0		0	
	3-4	1 (5.0)		2 (11.1)	
	4-5	19 (95.0)		13 (72.2)	
	No ratings	0		3 (16.7)	
**Number of ratings**					
	<100	5 (25.0)		1 (5.6)	
	100-1000	6 (30.0)		1 (5.6)	
	>1000	9 (45.0)		13 (72.2)	
	No ratings	0		3 (16.7)	

### Applicability of the Content of the Included Apps on Older Adults’ Depression Self-Care Needs

**Figure 2:**
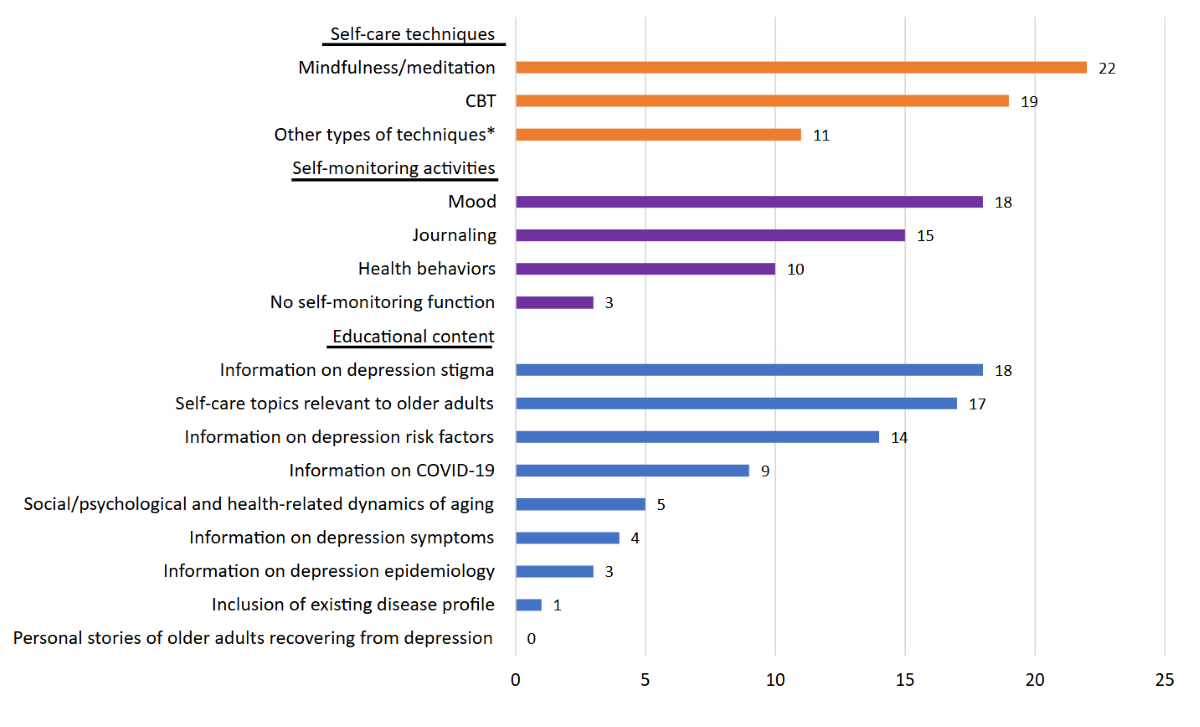
Content related to older adults. *Other types of techniques included acceptance and commitment therapy (n=4, 17.4%), behavioral activation (n=2, 8.7%), positive psychology (n=2, 8.7%), interpersonal therapy (n=2, 8.7%), problem-solving therapy (n=1, 4.3%), music therapy (n=1, 4.3%), and dialectical behavior therapy (n=1, 4.3%). In addition, 2 (8.7%) apps offered 2 types of techniques other than mindfulness/meditation and CBT, respectively; CBT: cognitive behavioral therapy.

Figure 2 presents a summary of app content related to older adults. All apps mainly targeted the general population instead of older adults. The most common self-care technique was mindfulness or meditation (n=22, 95.7%), while 19 (82.6%) apps used CBT. Other types of techniques included acceptance and commitment therapy (n=4, 17.4%), behavioral activation (n=2, 8.7%), positive psychology (n=2, 8.7%), interpersonal therapy (n=2, 8.7%), problem-solving therapy (n=1, 8.7%), music therapy (n=1, 4.3%), and dialectical behavior therapy (n=1, 4.3%). In addition, 2 (8.7%) apps offered 2 types of techniques other than mindfulness/meditation and CBT. More than half (n=17, 73.9%) of the included apps mentioned topics relevant to older adults, such as pain management, grief, loneliness, and social isolation. Most of the apps (n=20, 87%) provided self-monitoring of mood or allowed users to keep journals, while 10 (43.5%) apps asked users to track other behaviors, such as exercise, diet, sleep, and medication, in addition to mood and journaling. Each app’s self-care techniques and self-monitoring activities are shown in Multimedia Appendix 2.

Furthermore, 3 (13%) apps described depression epidemiology in older adults, 2 (66.7%) of which provided this information in conversations with generative artificial intelligence (AI) chatbots. In addition, 14 (60.9%) of the apps reported common risk factors of depression in older adults, such as chronic diseases, loss of family members, and loneliness, and 4 (17.4%) apps described depressive symptoms in older adults. Regarding the psychosocial and health-related dynamics of aging, 5 (21.7%) apps discussed how aging impacts life. When creating a new account in 1 (4.3%) app, there was a question about what diseases users have. More than two-thirds of the apps (n=18, 78.3%) addressed the stigma of depression by normalizing mental disorders, providing affirmations to users, and encouraging them to acknowledge and accept the illness. However, none of the apps shared a personal story of an older adult recovering from depression. Multimedia Appendix 3 shows the total score of each app on the 9 items of educational content. The mean score was 2.7 (range 1-5), indicating the scarcity of content relevant to older adults.

**Figure 3:**
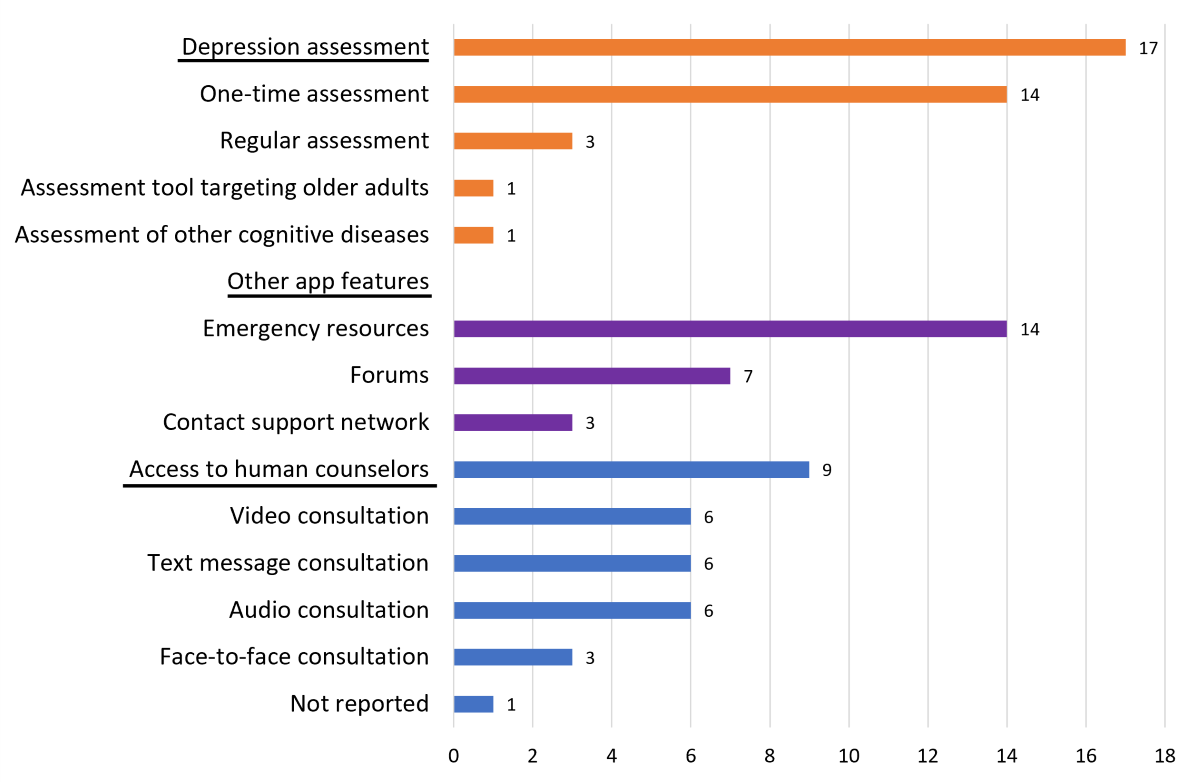
Main features of the apps included in this study.

Figure 3 presents the main features of the included apps, such as the assessment of depression, comorbid diseases, etc. Of the 23 apps, 17 (73.9%) provided depression assessment using validated tools, such as the Patient Health Questionnaire-9 (n=7, 41.2%). One Chinese app used the Geriatric Depression Scale, an assessment scale targeted at older adults, and also provided assessment tools for dementia, cognitive impairment, and functional ability (activities of daily living). Of the 17 (73.9%) apps with depression assessment tools, 14 (82.4%) administered the assessment once, while the other 3 (17.6%) offered regular assessment.

Furthermore, 7 (30.4%) apps had forums that allowed users to communicate with each other, and 9 (39.1%) apps provided access to counselors using video or audio calls, text messages, or emails. On 3 (75%) of the 4 (17.4%) Chinese apps, users could book face-to-face appointments with a counselor. The process for accessing counselors could not be evaluated on 1 (5.3%) English app because the feature was limited to India and we were Singapore based. In addition, 3 (13%) of the 23 apps allowed users to contact or seek help from their social network directly through the apps by entering the contact details of those people, while 14 (60.9%) apps included emergency resources, such as suicide prevention helplines, and users could be redirected to these services from the apps. Multimedia Appendix 4 shows each app’s main features.

### Comparison of Chinese and English Apps Based on Content

There were similarities and differences between the 4 (17.4%) Chinese and 19 (82.6%) English apps. The similarities were the inclusion of self-care techniques (eg, CBT and mindfulness, mood tracking, and journaling functions) and the reporting of depression risk factors in older adults. All English apps targeted users with mental health needs, but 3 (75%) of the 4 (17.4%) Chinese apps also included educational content for mental health providers. Perhaps for this reason, the Chinese apps contained more information (eg, mental disorder encyclopedia) and functions (eg, cognitive function assessment) than the English apps. Although all the included apps were free to download, 12 (63.2%) English apps had a paid version, where users could access all content in the apps with monthly or yearly subscriptions, while 1 (5.3%) English app and 3 (75%) Chinese apps adopted a different commercial mode by offering specific courses or assessments addressing various mental health issues with separate purchases.

### Applicability of the Technical Features of the Included Apps to Older Adults’ Needs

All apps offered some degree of personalization, including addressing the user by name, recording their preferences for dark mode and haptic feedback, and providing tailored suggestions and individualized responses from AI-assisted chatbots. Of the 23 apps, 15 (65.2%) had notifications to remind users to use the apps. In addition, 21 (91.3%) apps included information about contacting customer support in the case of technical issues, questions, or suggestions. Multimedia Appendix 5 shows detailed information about these technical features of each app.

Table 4 shows the selected items of MARS and mHONcode. Detailed information about these items of each app can be found in Multimedia Appendix 6. Using MARS, both involvement and aesthetics had a mean score of 3.8 (SD 0.4) out of 5.0. The average score for information credibility was 2.9 (SD 0.5) out of 5. Furthermore, 6 (26.1%) apps were evaluated in feasibility studies [86-88] or randomized controlled trials (RCTs) [89-91] and had positive outcomes related to participants’ mental health. None of the Chinese apps has yet been evaluated. However, the study participants mainly targeted the general adult population aged 18 years and above [86,87,89,91,92]. With regard to mHONcode, all but 3 (13%) apps had a privacy and confidentiality policy. Of the 20 (87%) apps with a privacy and confidentiality policy, 2 (10%) had inaccessible policies.

**Table 4:** Selected items of MARS^a^ and mHONcode^b^.

Technical features		Score (out of 5)
**MARS, mean (SD)**		
	Engagement	3.8 (0.4)
	Aesthetics	3.9 (0.6)
	Information credibility	2.9 (0.5)
	Evidence base of information	3.2 (1.0)
**mHONcode, n (%)**		
	Privacy and confidentiality policy	20 (87.0)
	Privacy policy accessible within the app	18 (78.3)

^a^MARS: Mobile Application Rating Scale.

^b^mHONcode: mobile Health On the Net Code.

### Accessibility Features

**Figure 4:**
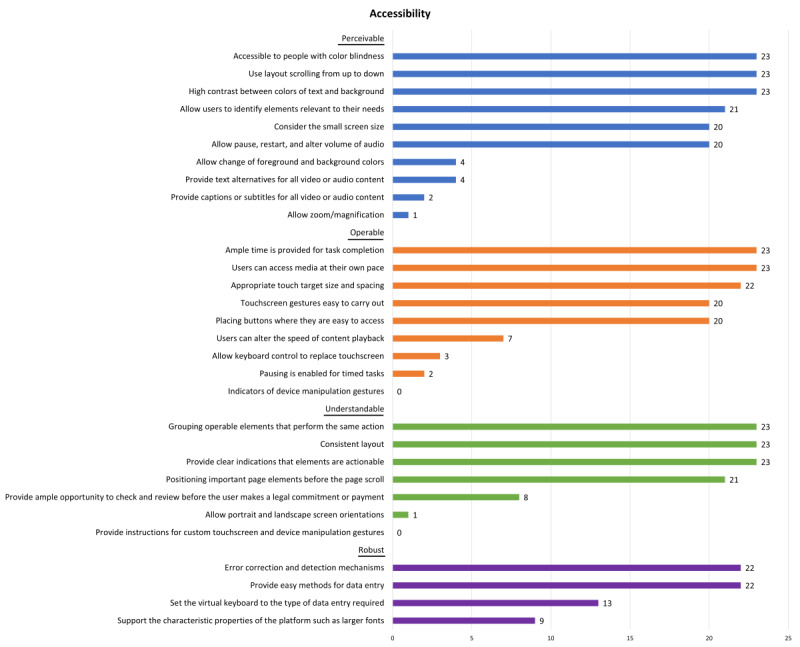
Accessibility of the apps included in this study.

Multimedia Appendix 7 shows each app’s accessibility feature and the total score. The mean percentage of each app’s score over its total score was 65.5% (range 58.6%-77.8%), suggesting moderate accessibility and areas for improvement. Figure 4 shows the accessibility of the included apps. Most of the apps (n=20, 87%) considered the smartphone’s small screen size and avoided delivering too much information on 1 page. The contrast between the text and the background color was visible in all apps (n=23, 100%), and 4 (17.4%) apps allowed users to change the foreground and background colors. All apps could be viewed by scrolling from top to bottom. Users would be able to identify elements relevant to their needs, and the content was accessible to people with color blindness. However, only 1 (4.3%) app allowed zooming or magnification. Of the 20 apps with video or audio content, a small proportion (n=4, 20%) provided text alternatives, while 2 (10%) provided captions or subtitles for all video and audio content. In addition, only 1 AI chatbot, Hector, could read out responses and provided users with the option to call rather than type.

The apps were generally easy to operate (ie, with an appropriate touch target size and spacing and easy touchscreen gestures). When connected to a Bluetooth keyboard, 3 (13%) of the 23 apps could be fully controlled by the keyboard, while 20 (87%) apps were partially or totally unresponsive to keyboard commands. Most of the apps did not restrict the time for completing a task, and 2 (50%) of 4 (17.4%) apps with a timed task allowed users to pause. Users could change the content playback speed in embedded videos in 7 (30.4%) apps, including YouTube videos in 4 (57.1%) apps.

All apps had a consistent layout and easy-to-recognize actionable buttons. Most of the apps (n=21, 91.3%) showed important elements regardless of page scroll. Although none of the apps provided instructions for custom touchscreen or device manipulation gestures, 3 (13%) apps from one company (Excel At Life) included a detailed tutorial explaining the meaning of each icon. Only 1 (4.3%) app allowed both portrait and landscape screen orientations, and 8 (34.8%) apps provided ample opportunity for users to check the terms and conditions and review them before making a payment.

Data entry on the apps was easy, supported by the error correction feature of the system’s default keyboard, and 13 (56.5%) apps provided user-friendly data entry methods by adjusting the keyboard according to the type of data. For example, a keyboard with “@” and “.com” was provided for entering email addresses. Lastly, although the phone’s operating system allows for fonts to be adjusted, only 9 (39.1%) apps supported this feature, with an additional app having adjustable font sizes within itself.

## Discussion

### Principal Findings

This systematic assessment of mental health apps is the first to have evaluated the applicability of existing mental health apps for depression self-care in older adults. We identified a total of 23 apps on Chinese and English app markets, but none of them specifically targets older adults with depression. A few apps provide information about depression specific to older adults or incorporated assessment tools for cognitive disorders or functional capacity. The level of accessibility, such as adaptations for users with visual or hearing impairments and adjustable font sizes, remains low, potentially limiting older adults’ use of the apps.

There is a lack of mental health apps to assist depression self-care in older adults. This gap in the current app market is somewhat at odds with other health care areas, as there are mobile health apps with medication reminders, apps for chronic disease management, apps promoting physical activity, and apps providing cognitive training activities [93]. Research has shown that a lack of consideration for older adults’ needs may reduce their perceived relevance and motivation to use such mobile apps [33]. However, a systematic review in 2023 showed that chatbots were promising in improving older adults’ depression and insomnia symptoms [94]. Although the use of generative AI chatbots in health care is still at an early stage, and its safety remains untested [95], the development of specific content for older adults must not be neglected.

We found fewer interactive Chinese depression self-care apps than English apps. Two recent systematic assessments of mental health apps in China identified 67 apps [76] and 172 apps [96]. These apps connected users with health care providers or provided noninteractive mental health education, but they frequently lacked self-care techniques [96] and thus were excluded from our study. Furthermore, despite the management of severe mental disorders being included in basic public health services in China, there are persisting challenges due to the lack of psychiatric professionals [97] and the traditional stigma associated with mental disorders [98]. Therefore, more interactive Chinese applications with self-care techniques are needed to support Chinese older adults’ mental health care demands.

We documented potential accessibility issues for older adults in the apps identified. According to qualitative studies on older adults’ experience with digital mental health interventions, the lack of detailed instructions is a barrier to their use [78,83]. Older adults have less experience with digital technology and are, at times, anxious about how to use it [99]. Therefore, clear instructions and guidance on how to use mobile apps are essential for many older adults, but we found only 3 of the 23 apps included a detailed tutorial for app navigation, suggesting a widespread lack of consideration for users with less technology literacy. Worldwide, around 1 in 10 adults over 50 years had a visual impairment in 2019 [100]. Hearing loss is also common in older adults, affecting over 50% of older males and 30% of older females aged 65 years and above [99]. Therefore, digital health interventions need to cater to their needs. We found that few apps with video or audio content provide text substitutes or captions or offer adjustable font sizes or are compatible with the accessibility features of larger text in the phone settings.

Around 30% of the depression self-care apps had an online forum, which may help reduce loneliness and increase social connections. A recent editorial highlighted the impact of loneliness and social isolation on adverse mental health outcomes, such as depression and anxiety [101]. Due to physical impairments, older adults are at a higher risk of loneliness and social isolation [102]. Online communities or forums could bring people with similar interests or circumstances together and provide them with a convenient opportunity to communicate from the comfort of their homes. A scoping review in 2022 found that online social networking improves older adults’ mental health and well-being by enhancing communication with their existing social network, increasing life satisfaction, and reducing depressive symptoms [103]. These communities may address the absence of interpersonal interactions in digital interventions by allowing older adults to provide each other emotional support, such as affirmation and encouragement, and technical advice to use developing technology [103-105].

### Strengths and Limitations

We used a consistent and reliable approach to app identification and assessment. We performed the search in Apple’s App Store, Google Play, and various Chinese Android app markets and adopted an AI screening tool that helped increase the efficiency by screening one-third of the names and descriptions of apps. The AI tool’s ability to retrieve 95% of the eligible records by screening 8%-33% of the total records was validated in simulation studies [49]. To capture all eligible apps, we further supplemented the search using Google and Baidu search engines. Two reviewers independently performed an in-depth assessment of apps by downloading and testing them on research phones. Second, there was no validated scale for the assessment of mobile apps for depression self-care in older adults. We developed an assessment rubric by synthesizing evidence from the extensive literature, including established clinical practice guidelines, systematic reviews, scoping reviews, academic books, and widely used guidelines or checklists of technical features.

However, this study also has some limitations. First, older adults with lived experience were not involved in the assessment, which calls for public and patient involvement in future studies targeting older adults. Another potential weakness is the exclusion of apps focused on well-being without explicitly addressing depression: we acknowledge that they might still be useful for older adults with mental disorders. However, the provision of information about depression is perhaps superior as it helps older adults gain awareness and knowledge [34] and reduces mental health stigma [106]. Third, we did not include apps in other languages due to the limited capacity of research manpower and resources.

The study has limited implications for clinical application in older adults because we did not identify any existing apps with high applicability to older adults. However, there are many implications for app developers and researchers in the field of digital mental health. As some older adults were skeptical of the credibility of apps [84], it is crucial for apps to clearly demonstrate their validity, reliability, and accuracy of information to enhance older adults’ trust. Three of the apps reviewed were clearly evaluated in the form of an RCT [89-91], and others may have been, but there was no mention of authors and sources, weakening the trust, real or perceived, in the evidence base of the existing apps. Although apps cannot replace clinical diagnosis and treatment, and users need to seek professional advice when necessary, self-care apps may still serve as an interim solution for older adults when they are waiting for clinical appointments [107], thereby providing them with early access to mental health resources and promoting their mental health. Future studies should aim to evaluate the clinical effectiveness of apps and their potential to lessen the strain on health care professionals.

### Conclusion

Although there are many depression self-care apps available in English and Chinese markets, our study suggests that they have limited applicability to older adults. The apps integrating generative AI chatbots have higher applicability because of tailored feedback and the provision of a text-to-speech option. In the future, new and updated depression self-care apps should consider incorporating content relevant to older adults, including online communities to reduce their social isolation, and improving accessibility for older adults with physical and sensory impairments.

## Appendix

Multimedia Appendix 1 Data extraction form.

Multimedia Appendix 2 Self-care techniques and self-monitoring activities of each app.

Multimedia Appendix 3 Educational content of each app.

Multimedia Appendix 4 Main features of each app.

Multimedia Appendix 5 Technical features of each app.

Multimedia Appendix 6 Selected items of MARS (Mobile Application Rating Scale) and mHONcode (mobile Health On the Net Code) for each app.

Multimedia Appendix 7 Accessibility of each app.

## Abbreviations

AI: artificial intelligence
CBT: cognitive behavioral therapy
MARS: Mobile Application Rating Scale
mHONcode: mobile Health On the Net Code
RCT: randomized controlled trial
W3C: World Wide Web Consortium

## Data Availability

The data supporting the findings of this study are available within the paper and its Multimedia Appendices.
