# Supplementary material for: Depression Self-Care Apps’ Characteristics and Applicability to Older Adults: Systematic Assessment
Source: J Med Internet Res. 2025 Feb 21;27:e56418. doi: 10.2196/56418 (PMC11890144; doi:10.2196/56418)
Supplement: Multimedia Appendix 1 [file jmir_v27i1e56418_app1.docx]

# **Multimedia Appendix 1**

# 1. Data extraction form for general features

| App ID | Numeric |
| --- | --- |
| App name | Free text |
| Developer name | Free text |
| Affiliation | Unknown/ Commercial/ Government/ NGO/ University |
| Platform | Android/iOS |
| When was the latest update | Free text |
| Version number of the app during the assessment | Free text |
| Rating on iOS | Numeric on a scale of 5 |
| Number of ratings | Numeric |
| Rating on Google Play | Numeric on a scale of 5 |
| Number of ratings | Numeric |
| Cost - basic version | Numeric |
| Cost - upgrade version (if any) | Numeric |
| Category in the app store | Health & fitness/ Medical/ Lifestyle |
| Total downloads in Android | Numeric |
| Languages | Free text |
| Target aspect of mental health | Free text |
| Privacy policy | Free text |
| What technical aspects are included in the app? | Allows sharing (Facebook, Twitter, etc.) |
|  | Allows password-protection |
|  | Requires login |
|  | Needs web access to function |
|  | Includes user profile |
|  | Allows exporting of data |
|  | Allows search within the app |

# 2. Data extraction form for content relevant to older adults

| **Field and description** | **Justification/Evidence** | **Options** |
| --- | --- | --- |
| **Symptoms and natural history of depression** |  |  |
| Does the app report epidemiological data on depression in older adults? | "Depression is the most common mental disorder in community settings and is a major cause of disability across the world…" [1-4] | Yes/No |
| Does the app report risk factors for depression? |  | Yes/No |
| If yes, what risk factors are reported? | Life stressors, loneliness, living in long-term care facilities, being a caregiver, and gender differences [5,6] | Free text |
| Does the app report risk factors that are more prevalent in older adults? | pain, loneliness, living in long-term care facilities, and being a caregiver [5,6] | Yes/No |
| If yes, what are the risk factors more prevalent in older adults? |  |  |
| Does the app introduce social/psychological and health-related dynamics of the aging process? | [7] | Yes/No |
| Does the app list the symptoms of depression in older adults? |  | Yes/No |
| Does the app explain relapse and recurrence of depression? | "The recurrence rate for depression is higher in elderly patients than in nonelderly patients" [8] | Yes/No |
| Does the app mention the risk of mild cognitive impairment, dementia, and Alzheimer's disease as a prognosis if not treated? | "Because depressive symptoms in elderly people may be a prodromal feature of dementia,36 and depressive episodes may be associated with an increased risk of dementia,37,38 progression to dementia should be carefully considered." [8] |  |
| Does the app ask patients about existing diseases, esp. cognitive impairment/dementia? | [7] | Yes/No |
| If yes, which diseases are included in the choice or can the user type? |  | Free text |
| Does the app address stigma in depression and mental health disorders in general? | "The assessment and treatment of major depressive disorder should consider … degree to which psychiatric illness is stigmatized" [3] "The stigma associated with depression cannot be ignored…" "When working with people with depression and their families or carers ... be aware that stigma and discrimination can be associated with a diagnosis of depression" [2] | Yes/No |
| Does the app address the importance of seeking help for people suffering from depression? | The stigma associated with mental health problems … may partly account for the reluctance of people with depression to seek help "People may have beliefs that prevent them from seeking help for depression…" [2] | Yes/No |
| Does the app include personal stories of older people recovering from depression? | “…interventions with social contact or first-person narratives were more effective than others…” [9] | Yes/No |
| **Screening of depression** |  |  |
| Does the app include any self-reported screening questionnaires? | "A range of self-rated and observer-rated questionnaires are available for the evaluation of depression…" [1] "If a person answers 'yes' to either of the depression identification questions (see 1.3.1.1), a practitioner who is competent to perform a mental health assessment should review the person's mental state..." "When assessing a person with suspected depression, consider using a validated measure... to inform and evaluate treatment..." [2] | Yes/No |
| If yes, which tool is used to screen depression, e.g., PHQ-9, CES-D, or Geriatric Depression Scale (GDS)? | [10] | Free text |
| Is the screening tool specific to older adults? |  | Yes/No |
| If the questionnaire suggests the user may be depressed, does the app suggest consulting an healthcare professional? | "A range of self-rated and observer-rated questionnaires are available for the evaluation of depression…" [1] "If a person answers 'yes' to either of the depression identification questions (see 1.3.1.1), a practitioner who is competent to perform a mental health assessment should review the person's mental state..." "When assessing a person with suspected depression, consider using a validated measure... to inform and evaluate treatment..." [2] | Yes/No |
| Does the app differentiate depression and dementia symptoms (apathy)? | [8] | Yes/No |
| If yes, which tool is used to screen apathy? | E.g., the Apathy Evaluation Scale and the Apathy Scale [8] | Free text |
| Does the app evaluate cognitive ability? | [7] | Yes/No |
| If yes, which tool is used to evaluate cognitive ability? |  | Free text |
| Does the app evaluate functional ability? | [7] | Yes/No |
| If yes, which tool is used to evaluate functional ability? |  | Free text |
| Is the depression screening tool administered regularly? | "It is important to regularly evaluate the patient’s general condition and treatment/care as needed when treating late-life depression." [8] |  |
| If yes, how often is the screening? |  |  |
| **Self-care techniques** |  |  |
| Does the app suggest lifestyle changes to manage depression? | "Patient education also includes general promotion of healthy behaviours such as exercise, good sleep hygiene, good nutrition, and decreased use of tobacco, alcohol, and other potentially deleterious substances" [1,3,4] | Yes/No |
| If yes, what lifestyle factors? |  |  |
| What type of self-care techniques does the app use? | CBT, interpersonal therapy, cognitive analytic therapy, problem-solving therapy, psychodynamic therapy, and mindfulness-based therapy [11,12] | Free text |
| What are the **topics** relevant to older adults that self-care techniques cover? | grief, pain management, loneliness |  |
| Does the app provide any self-monitoring tracking functions (e.g., mood, behaviour, journaling, or others that require user input)? |  | Yes/No |
| If yes, what types of active tracking functions are employed by the app? (choose all that apply) | Mood/Behaviour/Clinical Symptom/Others (specify) | Mood/Behaviour/Clinical Symptom/Others (specify) |
| **Personalisation** |  |  |
| Does the app have any features which promote personalisation? | E.g., timings, record a history of user's preferences/health status, goals etc. [13] | Yes/No |
| If yes, what personalisation features? |  | Yes/No |
| Does the app offer reminders? | [14] | Yes/No |
| If yes, how often can the reminder be set? |  | Free text |
| Can the pace of the therapy be personalised? | [7] | Yes/No |
| Does the app use age-appropriate and common languages? | [15,16] | Yes/No |
| **Human involvement** |  |  |
| Does the app include information to access counsellors for the users? | [17] | Yes/No |
| If yes, in what way can a counsellor be contacted? | phone/ video/ text message/ online chat | Free text |
| Does the app allow users to share information or contact people in her/his support network? | E.g., family members, friends, support groups, others | Yes/No |
| Does the app allow users to join discussion forums? | "Social support has also been investigated as a risk factor for development of late-life depression." [10] | Yes/No |
| **Other functions** |  |  |
| Does the app assess users’ risk of suicide? | "It is also critical to determine the degree to which patients might be suicidal" [18] | Yes/No |
| Does the app allow the user to contact emergency services directly through the app? |  | Yes/No |
| If yes, please list the resources offered by the app | “always ask people with depression directly about suicidal ideation and intent. If there is a risk of self-harm or suicide: assess whether the person has adequate social support and is aware of sources of help” [2] | Free text |
| Is there a helpline specifically for older adults? | [19] | Yes/No |
| Does the app provide information about mental health issues during the COVID-19 pandemic? | [17] | Yes/No |
| Does the app inform the users how often they should use the app? | [13] | Yes/No |
| Does the app offer other functions to users? |  | Yes/No |
| If yes, what functionalities does the app offer? |  | Free text |

3.1 Mobile Application Rating Scale

| **MARS** |  |  |
| --- | --- | --- |
| Engagement – fun, interesting, customisable, interactive (e.g. sends alerts, messages, reminders, feedback, enables sharing), well-targeted to audience | Target group: Is the app content (visual information, language, design) appropriate for your target audience? | 1 Completely inappropriate/ unclear/confusing 2 Mostly inappropriate/unclear/ confusing 3 Acceptable but not targeted. May be inappropriate/unclear/ confusing 4 Well-targeted, with negligible issues 5 Perfectly targeted, no issues found |
| Aesthetics – graphic design, overall visual appeal, colour scheme, and stylistic consistency | Graphics: How high is the quality/resolution of graphics used for buttons/icons/menus/ content? | 1 Graphics appear amateur, very poor visual design - disproportionate, completely stylistically inconsistent 2 Low quality/low resolution graphics; low quality visual design – disproportionate, stylistically inconsistent 3 Moderate quality graphics and visual design (generally consistent in style) 4 High quality/resolution graphics and visual design – mostly proportionate, stylistically consistent 5 Very high quality/resolution graphics and visual design - proportionate, stylistically consistent throughout |
| Information – Contains high quality information (e.g. text, feedback, measures, references) from a credible source. Select N/A if the app component is irrelevant. | Credibility: Does the app come from a legitimate source (specified in app store description or within the app itself)? | 1 Source identified but legitimacy/trustworthiness of source is questionable (e.g. commercial business with vested interest) 2 Appears to come from a legitimate source, but it cannot be verified (e.g. has no webpage) 3 Developed by small NGO /institution (hospital/centre, etc.) /specialised commercial business, funding body 4 Developed by government, university or as above but larger in scale 5 Developed using nationally competitive government or research funding (e.g. Australian Research Council, NHMRC) |
|  | Evidence base: Has the app been trialled/tested; must be verified by evidence (in published scientific literature)? | N/A The app has not been trialled/tested 1 The evidence suggests the app does not work 2 App has been trialled (e.g., acceptability, usability, satisfaction ratings) and has partially positive outcomes in studies that are not randomised controlled trials (RCTs), or there is little or no contradictory evidence. 3 App has been trialled (e.g., acceptability, usability, satisfaction ratings) and has positive outcomes in studies that are not RCTs, and there is no contradictory evidence. 4 App has been trialled and outcome tested in 1-2 RCTs indicating positive results 5 App has been trialled and outcome tested in > 3 high quality RCTs indicating positive results |

**3.2 Mobile Health On the Net Code**

| **Confidentiality** |  |
| --- | --- |
| Is there a privacy and confidentiality clause in the app? | Yes/No |
| Is the privacy policy easily accessible within the app? | Yes/No |
| Is consent to data collection required at the first launch of the application? | Yes/No |
| Where the data is stored (on the application, on the SD card, etc.) | Free text |
| Is the data transmitted to third parties? | Yes/No |
| If yes, to which third parties are they transmitted? | Free text |

**3.3 Mobile Accessibility: How WCAG 2.0 and Other W3C/WAI Guidelines Apply to Mobile and Guidance on Applying WCAG 2.0 to Non-Web Information and Communications Technologies (WCAG2ICT)**

| **Principle 1: Perceivable** | | |
| --- | --- | --- |
| Small Screen Size | Is the amount of information appropriate on the screen? Does the app provide a reasonable default size for content and touch controls? | Yes/No |
| Zoom/Magnification | Does the app allow users to magnify the screen? | Yes/No |
| Contrast | Is the contrast of different levels of text visible? For example, a contrast ratio of 1.2 times bold or 1.5 times of the default platform size | Yes/No |
| Non-Linear Screen Layouts | Does the app use the layout scrolling from up to down? If not, what are the layoutes? For example, starting from the middle? | Yes/No |
| Text Alternatives | Users can access a text alternative that serves an equivalent purpose for all non-text content (e.g. images, video) | Yes/No |
|  | Closed Captions or Subtitles are included for all video and audio content | Yes/No |
| Distinguishable | Users can identify which elements are most pertinent for their needs. | Yes/No |
|  | Color is not the sole means used to convey meaning, structure, or purpose of content | Yes/No |
|  | For blocks of text, users can select appropriate foreground and background colors | Yes/No |
|  | If any audio on a web page plays automatically, a mechanism to pause, restart, or alter content volume is provided | Yes/No |
| **Principle 2: Operable** |  |  |
| Keyboard Control for Touchscreen Devices | Does it allow mobile devices to be operated using external physical keyboards (e.g. keyboards connected via Bluetooth, USB On-The-Go) or alternative on-screen keyboards (e.g. scanning on-screen keyboards)? | Yes/No |
| Touch Target Size and Spacing | Are touch targets larger than 9*9mm with enough inactive space in between? | Yes/No |
| Touchscreen Gestures | Are touchscreen gestures easy to carry out? Are there indicators of gestures? | Yes/No |
| Device Manipulation Gestures | Are there device manipulation gestures (e.g. shaking or tilting)?  If yes, 1) are there features from an onscreen menu that allow users to work-around the device manipulation gestures? 2) are there onscreen indicators that remind people how and when to use them? | Yes/No |
| Placing buttons where they are easy to access | Are buttons easy to reach when the device is held in different positions using one hand only? Is it easy to access using left or right thumb? | Yes/No |
| Enough Time | Users can access media at their own pace | Yes/No |
|  | Ample time is provided for task completion, and where applicable, users can turn off a time limit before encountering it | Yes/No |
|  | Pausing is enabled for timed tasks | Yes/No |
|  | Users can alter speed of content playback | Yes/No |
| **Principle 3: Understandable** | | |
| Changing Screen Orientation (Portrait/Landscape) | Does the app support both portrait and lanscape orientations? | Yes/No |
| Consistent Layout | Are components repeated across multiple pages presented in a consistent layout? | Yes/No |
| Positioning important page elements before the page scroll | Is important page information visible without having to scroll the view? | Yes/No |
| Grouping operable elements that perform the same action | Does the app group operable elements that perform the same action or go to the same destination (e.g. link icon with link text)? | Yes/No |
| Provide clear indication that elements are actionable | Does the app provide a clear indication that elements are actionable? For example, button shape, check box, home icon, different colors, underlined text for links | Yes/No |
| Provide instructions for custom touchscreen and device manipulation gestures | Does the app provide instructions for **custom** touchscreen and device manipulation gestures? For example, some computer games allow users to determine the characters to move instead of WASD; rolling up/down | Yes/No |
| Input Assistance | For apps that cause legal commitment or financial transactions: Submissions are reversible (where possible) and given ample opportunity to be checked and reviewed by the user | Yes/No |
| **Principle 4: Robust** | | |
| Set the virtual keyboard to the type of data entry required | Does the app provide different virtual keyboards depending on the type of data entry? For example, number keyboard (9 key), email address with "@" or ".com" | Yes/No |
| Provide easy methods for data entry | Is data entry easy, such as having select menus, check boxes, or automatically entering known information (e.g. date, time, location)? | Yes/No |
| Input Assistance | Error-correction and detection mechanisms are used to assist users entering information to forms or other input points. Predictive text for input fields to help offer alternatives when errors in user input are detected | Yes/No |
| Support the characteristic properties of the platform | Does the app support the characteristic properties of the platform? For example, most platforms have the ability to set large fonts, but not all applications honor it for all text. | Yes/No |

## References

1. Ministry of Health Singapore. *Depression - MOH Clinical Practice Guidelines 1/2012*. Singapore: Ministry of Health; 2012.
2. NICE. *Depression in Adults: Recognition and Management*. London, UK: National Institute for Health and Care Excellence; 2009.
3. American Psychiatric Association. *Practice Guideline for the Treatment of Patients with Major Depressive Disorder. 3rd ed*. Arlington, VA: American Psychiatric Association; 2010.
4. Malhi GS, Bassett D, Boyce P, et al. Royal Australian and New Zealand College of Psychiatrists clinical practice guidelines for mood disorders. *Aust N Z J Psychiatry* 2015;49(12):1087-1206.
5. Maier A, Riedel-Heller SG, Pabst A, Luppa M. Risk factors and protective factors of depression in older people 65+. A systematic review. *PLoS One* 2021 May 13;16(5):e0251326. doi: 10.1371/journal.pone.0251326.
6. Laidlaw, K., McAlpine, S. Cognitive behaviour therapy: how is it different with older people?. *J Rat-Emo Cognitive-Behav Ther* 2008;26:250-262. https://doi.org/10.1007/s10942-008-0085-6
7. Practice guideline for the treatment of patients with major depressive disorder (revision). *American Journal of Psychiatry* 2000;157(4 SUPPL.).
8. Baba H, Kito S, Nukariya K, Takeshima M, Fujise N, Iga J, et al. Guidelines for diagnosis and treatment of depression in older adults: a report from the Japanese Society of Mood Disorders. *Psychiatry Clin Neurosci* 2022;76(6):222-234.
9. Thornicroft G, Mehta N, Clement S, et al. Evidence for effective interventions to reduce mental-health-related stigma and discrimination. *Lancet* 2016;387(10023): 1123-1132.
10. Pachana NA, Laidlaw K (eds). *The Oxford Handbook of Clinical Geropsychology*. Oxford, UK: Oxford University Press; 2014. https://doi.org/10.1093/oxfordhb/9780199663170.001.0001.
11. Hepple J. Psychotherapies with older people: an overview. *Adv Psychiatr Treat* 2004;10(5):371-377. doi:10.1192/apt.10.5.371.
12. Avasthi A, Grover S. Clinical practice guidelines for management of depression in elderly. *Indian J Psychiatry* 2018 Feb;60(Suppl 3):S341-S362. doi: 10.4103/0019-5545.224474.
13. Barriers and facilitators for the use of digital technologies for mental health by older adults: a qualitative systematic review (working paper).
14. Jakob R, Harperink S, Rudolf AM, Fleisch E, Haug S, Mair JL, et al. Factors influencing adherence to mHealth apps for prevention or management of noncommunicable diseases: systematic review. *J Med Internet Res* 2022;24(5):e35371.
15. Liu N, Yin J, Tan SS, Ngiam KY, Teo HH. Mobile health applications for older adults: a systematic review of interface and persuasive feature design. *J Am Med Inform Assoc* 2021 Oct 12;28(11):2483-2501. doi: 10.1093/jamia/ocab151.
16. Moody HR, Sasser, JR. *Aging: Concepts and Controversies, 9th edition*. Sage Publications; 2018.
17. Martinengo L, Stona AC, Griva K, Dazzan P, Pariante CM, von Wangenheim F, Car J. Self-guided cognitive behavioral therapy apps for depression: systematic assessment of features, functionality, and congruence with evidence. *J Med Internet Res* 2021 Jul 30;23(7):e27619. doi: 10.2196/27619.
18. Beck J. *Cognitive Behavior Therapy: Basics and Beyond, 2nd ed*. New York, NY. US: Guilford Press; 2011.
19. Wysa mobile application. https://www.wysa.io/ [accessed on 09/01/2023].
