# Supplementary material for: Depression Self-Care Apps’ Characteristics and Applicability to Older Adults: Systematic Assessment
Source: J Med Internet Res. 2025 Feb 21;27:e56418. doi: 10.2196/56418 (PMC11890144; doi:10.2196/56418)
Supplement: Multimedia Appendix 3 [file jmir_v27i1e56418_app3.docx]

**Appendix 3. Educational content in each app**

| Educational content | Information on depression stigma | Self-care topics relevant to older adults | Information on depression risk factors | Information on COVID-19 | Social/ psychological and health-related dynamics of ageing | Information on depression symptoms | Information on depression epidemiology | Inclusion of existing disease profile | Personal stories of older adults recovering from depression | Summed score/total score |
| --- | --- | --- | --- | --- | --- | --- | --- | --- | --- | --- |
| Youper: Self-Guided Therapy | No | Yes | No | No | No | No | No | No | No | 1/9 |
| Wysa: Mental Health Support | No | Yes | No | Yes | No | No | No | No | No | 1/9 |
| Sanvello: Anxiety & Depression | Yes | Yes | No | No | No | No | No | Yes | No | 3/9 |
| MindDoc: Your Companion | No | Yes | Yes | Yes | No | No | No | No | No | 2/9 |
| Hector: Mental Health Therapy | Yes | No | Yes | Yes | Yes | Yes | Yes | No | No | 5/9 |
| Mindspa: The Mental Health App | No | Yes | No | Yes | No | No | No | No | No | 1/9 |
| What's Up? A Mental Health App | Yes | No | No | No | No | No | No | No | No | 1/9 |
| Amaha: Mental Health Self-Care | Yes | Yes | Yes | Yes | No | No | No | No | No | 3/9 |
| SoundMind: Music Therapy | Yes | Yes | No | Yes | No | No | No | No | No | 2/9 |
| Feelmo: Mental Health Support | Yes | Yes | No | No | No | No | No | No | No | 2/9 |
| Happier You-Community, therapy | Yes | No | Yes | No | No | No | No | No | No | 2/9 |
| MyPossibleSelf: Mental Health | Yes | Yes | Yes | No | No | No | No | No | No | 3/9 |
| Happify | Yes | Yes | Yes | No | Yes | No | No | No | No | 4/9 |
| 7 Cups: Therapy & Support | Yes | Yes | No | No | Yes | No | No | No | No | 3/9 |
| Stop Panic & Anxiety Self-Help | Yes | Yes | Yes | No | No | No | No | No | No | 3/9 |
| CBT Thought Diary | Yes | Yes | Yes | No | No | No | No | No | No | 3/9 |
| CBT Guide to Depression & Test | Yes | Yes | Yes | No | No | No | No | No | No | 3/9 |
| CBT Tools for Healthy Living | Yes | Yes | Yes | No | No | No | No | No | No | 3/9 |
| CBT Therapy: Mental Healthcare | Yes | No | Yes | Yes | Yes | Yes | Yes | No | No | 5/9 |
| 简单心理 - 专业心理咨询 | Yes | No | Yes | Yes | No | Yes | No | No | No | 3/9 |
| 壹心理-心理情感咨询 | Yes | No | Yes | Yes | No | No | No | No | No | 2/9 |
| Now冥想 | Yes | Yes | No | No | No | No | No | No | No | 2/9 |
| 心理咨询壹点灵 | No | Yes | Yes | No | Yes | Yes | Yes | No | No | 5/9 |

For every item, a “Yes” was assigned one point.
