# Supplementary material for: Depression Self-Care Apps’ Characteristics and Applicability to Older Adults: Systematic Assessment
Source: J Med Internet Res. 2025 Feb 21;27:e56418. doi: 10.2196/56418 (PMC11890144; doi:10.2196/56418)
Supplement: Multimedia Appendix 4 [file jmir_v27i1e56418_app4.docx]

**Appendix 4. Main features of each app**

| App name | Depression assessment | Depression assessment frequency | Depression assessment tool targeting older adults | Assessment of other cognitive diseases | Other features | | |  |
| --- | --- | --- | --- | --- | --- | --- | --- | --- |
|  |  |  |  |  | Emergency resources | Forums | Contact support network | |
| Youper: Self-Guided Therapy | Yes | Regular | No | No | No | No | No | |
| Wysa: Mental Health Support | Yes | One-time | No | No | Yes | No | No | |
| Sanvello: Anxiety & Depression | Yes | Regular | No | No | Yes | Yes | Yes | |
| MindDoc: Your Companion | No | N/A | N/A | No | Yes | No | No | |
| Hector: Mental Health Therapy | No | N/A | N/A | No | No | No | No | |
| Mindspa: The Mental Health App | Yes | One-time | No | No | No | No | No | |
| What's Up? A Mental Health App | No | N/A | N/A | No | Yes | No | No | |
| Amaha: Mental Health Self-Care | Yes | One-time | No | No | No | Yes | No | |
| SoundMind: Music Therapy | Yes | One-time | No | No | Yes | No | Yes | |
| Feelmo: Mental Health Support | No | N/A | N/A | No | Yes | No | No | |
| Happier You - Community, therapy | Yes | One-time | No | No | Yes | Yes | No | |
| MyPossibleSelf: Mental Health | No | N/A | N/A | No | Yes | No | No | |
| Happify | No | N/A | N/A | No | Yes | Yes | No | |
| 7 Cups: Therapy & Support | Yes | One-time | No | No | Yes | Yes | No | |
| Stop Panic & Anxiety Self-Help | Yes | One-time | No | No | No | No | No | |
| CBT Thought Diary | Yes | One-time | No | No | No | No | No | |
| CBT Guide to Depression & Test | Yes | One-time | No | No | Yes | No | No | |
| CBT Tools for Healthy Living | Yes | One-time | No | No | No | No | No | |
| CBT Therapy: Mental Healthcare | Yes | One-time | No | No | No | No | No | |
| 简单心理 - 专业心理咨询 | Yes | Regular | No | No | Yes | No | No | |
| 壹心理-心理情感咨询 | Yes | One-time | No | No | Yes | Yes | Yes | |
| Now冥想 | Yes | One-time | No | No | No | No | No | |
| 心理咨询壹点灵 | Yes | One-time | Yes | Yes | Yes | Yes | No | |

Appendix 4. Main features of each app (continued)

| App name | Other features | | | | | |
| --- | --- | --- | --- | --- | --- | --- |
|  | Access to human counsellors | Video consultation | Text message consultation | Audio consultation | Face-to-face consultation | Communication methods not specified |
| Youper: Self-Guided Therapy | No | N/A | N/A | N/A | N/A | / |
| Wysa: Mental Health Support | Yes | No | Yes | Yes | No | / |
| Sanvello: Anxiety & Depression | No | N/A | N/A | N/A | N/A | / |
| MindDoc: Your Companion | Yes | Yes | Yes | Yes | No | / |
| Hector: Mental Health Therapy | No | N/A | N/A | N/A | N/A | / |
| Mindspa: The Mental Health App | No | N/A | N/A | N/A | N/A | / |
| What's Up? A Mental Health App | No | N/A | N/A | N/A | N/A | N/A |
| Amaha: Mental Health Self-Care | Yes | / | / | / | / | Yes |
| SoundMind: Music Therapy | No | N/A | N/A | N/A | N/A | N/A |
| Feelmo: Mental Health Support | Yes | No | No | Yes | No | / |
| Happier You - Community, therapy | Yes | Yes | Yes | No | No | / |
| MyPossibleSelf: Mental Health | No | N/A | N/A | N/A | N/A | N/A |
| Happify | No | N/A | N/A | N/A | N/A | N/A |
| 7 Cups: Therapy & Support | Yes | Yes | Yes | Yes | No | / |
| Stop Panic & Anxiety Self-Help | No | N/A | N/A | N/A | N/A | N/A |
| CBT Thought Diary | No | N/A | N/A | N/A | N/A | N/A |
| CBT Guide to Depression & Test | No | N/A | N/A | N/A | N/A | N/A |
| CBT Tools for Healthy Living | No | N/A | N/A | N/A | N/A | N/A |
| CBT Therapy: Mental Healthcare | No | N/A | N/A | N/A | N/A | N/A |
| 简单心理 - 专业心理咨询 | Yes | Yes | No | No | Yes | / |
| 壹心理-心理情感咨询 | Yes | Yes | Yes | Yes | Yes | / |
| Now冥想 | No | N/A | N/A | N/A | N/A | N/A |
| 心理咨询壹点灵 | Yes | Yes | Yes | Yes | Yes | / |

N/A: not applicable

For every item, a “Yes” was assigned one point.
