# Supplementary material for: Depression Self-Care Apps’ Characteristics and Applicability to Older Adults: Systematic Assessment
Source: J Med Internet Res. 2025 Feb 21;27:e56418. doi: 10.2196/56418 (PMC11890144; doi:10.2196/56418)
Supplement: Multimedia Appendix 5 [file jmir_v27i1e56418_app5.docx]

Appendix 5. Technical features of each app

| App name | Personalization | Reminders | Contact customer support |
| --- | --- | --- | --- |
|  |  |  |  |
| Youper: Self-Guided Therapy | Yes | Yes | Yes |
| Wysa: Mental Health Support | Yes | No | Yes |
| Sanvello: Anxiety & Depression | Yes | Yes | Yes |
| MindDoc: Your Companion | Yes | Yes | Yes |
| Hector: Mental Health Therapy | Yes | No | Yes |
| Mindspa: The Mental Health App | Yes | No | Yes |
| What's Up? A Mental Health App | Yes | No | Yes |
| Amaha: Mental Health Self-Care | Yes | Yes | No |
| SoundMind: Music Therapy | Yes | No | Yes |
| Feelmo: Mental Health Support | Yes | Yes | No |
| Happier You - Community, therapy | Yes | No | Yes |
| MyPossibleSelf: Mental Health | Yes | Yes | Yes |
| Happify | Yes | No | Yes |
| 7 Cups: Therapy & Support | Yes | Yes | Yes |
| Stop Panic & Anxiety Self-Help | Yes | Yes | Yes |
| CBT Thought Diary | Yes | Yes | Yes |
| CBT Guide to Depression & Test | Yes | Yes | Yes |
| CBT Tools for Healthy Living | Yes | Yes | Yes |
| CBT Therapy: Mental Healthcare | Yes | Yes | Yes |
| 简单心理 - 专业心理咨询 | Yes | Yes | Yes |
| 壹心理-心理情感咨询 | Yes | Yes | Yes |
| Now冥想 | Yes | Yes | Yes |
| 心理咨询壹点灵 | Yes | No | Yes |
