# Supplementary material for: Depression Self-Care Apps’ Characteristics and Applicability to Older Adults: Systematic Assessment
Source: J Med Internet Res. 2025 Feb 21;27:e56418. doi: 10.2196/56418 (PMC11890144; doi:10.2196/56418)
Supplement: Multimedia Appendix 6 [file jmir_v27i1e56418_app6.docx]

**Appendix 6. Technical features of each app**

| App name | Mobile Application Rating Scale (MARS) | | | | mobile Health On the Net Code (mHONcode) | |
| --- | --- | --- | --- | --- | --- | --- |
|  | Engagement | Asthetics | Information | | Has privacy and confidentiality policy | Privacy policy accessible within the app |
|  |  |  | Credibility | Evidence base |  |  |
| Youper: Self-Guided Therapy | 4 | 3 | 3 | 2 | Yes | No |
| Wysa: Mental Health Support | 4 | 4 | 3 | 3 | Yes | Yes |
| Sanvello: Anxiety & Depression | 4 | 4 | 3 | 4 | Yes | Yes |
| MindDoc: Your Companion | 4 | 5 | 3 | N/A | Yes | Yes |
| Hector: Mental Health Therapy | 3 | 4 | 3 | N/A | No | No |
| Mindspa: The Mental Health App | 4 | 4 | 3 | N/A | No | No |
| What's Up? A Mental Health App | 4 | 3 | 1 | N/A | Yes | No |
| Amaha: Mental Health Self-Care | 4 | 4 | 3 | N/A | Yes | Yes |
| SoundMind: Music Therapy | 3 | 4 | 3 | N/A | Yes | Yes |
| Feelmo: Mental Health Support | 4 | 4 | 3 | N/A | Yes | Yes |
| Happier You - Community, therapy | 4 | 4 | 3 | N/A | No | No |
| MyPossibleSelf: Mental Health | 4 | 4 | 3 | 4 | Yes | Yes |
| Happify | 4 | 4 | 3 | 4 | Yes | Yes |
| 7 Cups: Therapy & Support | 3 | 3 | 4 | 2 | Yes | Yes |
| Stop Panic & Anxiety Self-Help | 4 | 4 | 3 | N/A | Yes | Yes |
| CBT Thought Diary | 3 | 4 | 3 | N/A | Yes | Yes |
| CBT Guide to Depression & Test | 4 | 4 | 3 | N/A | Yes | Yes |
| CBT Tools for Healthy Living | 4 | 4 | 3 | N/A | Yes | Yes |
| CBT Therapy: Mental Healthcare | 4 | 5 | 3 | N/A | Yes | Yes |
| 简单心理 - 专业心理咨询 | 4 | 4 | 3 | N/A | Yes | Yes |
| 壹心理-心理情感咨询 | 4 | 4 | 3 | N/A | Yes | Yes |
| Now冥想 | 4 | 4 | 2 | N/A | Yes | Yes |
| 心理咨询壹点灵 | 3 | 2 | 3 | N/A | Yes | Yes |

N/A: not applicable

For every item in mobile Health On the Net Code (mHONcode), a “Yes” was assigned one point.
